# Tryptanthrin Protects Mice against Dextran Sulfate Sodium-Induced Colitis through Inhibition of TNF-α/NF-κB and IL-6/STAT3 Pathways

**DOI:** 10.3390/molecules23051062

**Published:** 2018-05-02

**Authors:** Zheng Wang, Xue Wu, Cui-Ling Wang, Li Wang, Chen Sun, Dong-Bo Zhang, Jian-Li Liu, Yan-Ni Liang, Dong-Xin Tang, Zhi-Shu Tang

**Affiliations:** 1 Shaanxi Collaborative Innovation Center of Chinese Medicinal Resources Industrialization, Shaanxi University of Chinese Medicine, Xianyang 712083, China; wazh0405@126.com (Z.W.); 1862927248@163.com(L.W.); sunchen2010xian@sina.com (C.S.); 2 Shaanxi Province Key Laboratory of New Drugs and Chinese Medicine Foundation Research, Shaanxi University of Chinese Medicine, Xianyang 712083, China; zhengshangxuew@163.com; 3 Shaanxi Rheumatism and Tumor Center of TCM Engineering Technology Research, Shaanxi University of Chinese Medicine, Xianyang 712083, China; 1501009@sntcm.edu.cn; 4 Key Laboratory of Resource Biology and Biotechnology in Western China, Ministry of Education, College of Life Science, Northwest University, Xi’an 710069, China; wangcl@nwu.edu.cn (C.-L.W.); jlliu@nwu.edu.cn (J.-L.L.); 5 Guizhou Province Hospital of Traditional Chinese Medicine, Guiyang University of Chinese Medicine, Guiyang 550002, China

**Keywords:** tryptanthrin, colitis DSS, NF-κBp65, STAT3

## Abstract

Inflammatory bowel disease (IBD) is a notable health problem and may considerably affect the quality of human life. Previously, the protective roles of tryptanthrin (TRYP) against dextran sulfate sodium (DSS) induced colitis has been proved, but the concrete mechanism remained elusive. It has been suggested that TRYP could diminish the weight loss and improve the health conditions of mice with DSS induced colitis. Hematoxylin and eosin staining revealed that TRYP could improve the histopathological structure of the colon tissue. Two signaling pathways (TNF-α/NF-κBp65 and IL-6/STAT3) were investigated using immunochemistry and western blot. The detected concentrations of the two cytokines TNF-α and IL-6 showed that their levels decreased after TRYP treatment of the colitis. The protein expression level of NF-κBp65 in cytoplasm increased after TRYP treatment of the induced colitis. However, the protein level of NF-κBp65 in the nucleus decreased after administration of TRYP. The expression level of IκBα, the inhibitory protein of NF-κBp65, was tested and the results suggested that TRYP could inhibit the degradation of IκBα. The phosphorylation level of STAT3 was inhibited by TRYP and the expression level of STAT3 and *p*-STAT3 decreased after administration of TRYP. We conclude that TRYP improves the health condition of mice with DSS induced colitis by regulating the TNF-α/NF-κBp65 and IL-6/STAT3 signaling pathways via inhibiting the degradation of IκBα and the phosphorylation of STAT3.

## 1. Introduction

Inflammatory bowel disease (IBD) is a complex set of non-specific intestinal inflammatory diseases with unknown etiology, including ulcerative colitis (UC) and Crohn’s disease (CD) that may eventually results in hyperplasia and neoplastic growth [1,2]. The main symptoms of this chronic disease are diarrhea, weight loss, abdominal pain, rectal bleeding, and vomiting [3]. The incidence of this disease in humans appears to be on the increase possibly due to a complex interaction of diet changes combined with genetic, immunoregulatory, and environmental factors [4]. For the pharmaceutical treatment of IBD anti-inflammatory agents and immunomodulators are used, but problems about their efficacy, safety and side-effects are reported with increasing frequency [5]. 

Though the etiology and pathogenesis of IBD have not been elucidated, the imbalances between anti-inflammatory cytokines (e.g., interleukin (IL)-11, IL-10, IL-4) and pro-inflammatory cytokines (e.g., IL-β, IL-6, IL-12, IL-17A, interferon-γ (IL-γ), and tumor necrosis factor-α (TNF-α)) are supposed to play an essential role in modulating the inflammation [6,7]. Moreover, the levels of pro-inflammatory cytokines IL-β, IL-6 and TNF-αare increased in dextran sulfate sodium (DSS)-induced colitis in mice. Accumulating experimental data indicates that these inflammatory factors are connected with some signaling pathways to modulate apoptosis and migration of cells, combined with the synthesis and secretion of some proteins [8,9]. Besides, it was reported that probiotic treatment could decrease NO production in plasma induced by DSS and this closely related with the expression of iNOS and NF-κB [10]. And it was interesting that a positive correlation between NO production and increased pro-inflammatory cytokine levels (TNF-α, IL-17 and IL-γ) were reported in patients with IBD [11]. Furtherly, it was reported that IL-23/IL-17A axis played a pivotal role in IBD pathogenesis through the NO pathway [12].

Nuclear factor-kappa B (NF-κB) is a key transcription factor that can be initiated by a vast array of stimuli relating to many biological process such as inflammation, immunity, differentiation, cell growth, apoptosis and tumorigenesis [13]. The NF-κB family consists of several proteins-p65 (RelA), RelB, c-Rel, p105/p50 (NF-κB1) and p100/52 (NF-κB2). These proteins can form homo- and heterodimeric complexes by associating each other with transcriptionally regulate target genes [14]. The signaling of NF-κB is normally suppressed by a family of inhibitory molecules termed IκB proteins, such as IκBα which binds to NF-κB to prevent its activation in the cytoplasm [15,16]. In addition, NF-κB plays a vital role in the pathogenesis of IBD, and the degree of activated NF-κB is significantly correlated with the severity of inflammation of the intestine and the colon [17]. 

Increasing evidence suggests that the signal transducer and activator of transcription 3 (STAT3) is an important intracellular signal transduction molecule because it is closely related to a number of inflammatory mediators and is normally present in the cytoplasm [18,19]. Once STAT3 is phosphorylated, it can translocate to the nucleus to regulate genes involved in apoptosis, migration, survival, etc. [20]. It is reported that IL-6 can activated STAT3 and has a significant role in the pathogenesis of IBD [21]. Therefore, an effective blockade of the signaling pathway is likely to be beneficial for the treatment of IBD. 

**Figure 1:**
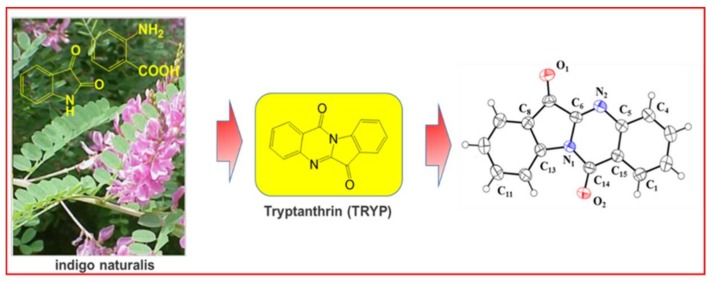
Chemical and monocrystal structures of TRYP.

Though treatments for IBD are available, their side effects and safety remain unknown. Traditional Chinese Medicine (TCM) is a treasure trove gifted by Nature for discovering novel compounds. TCM has proved to be effective clinically for thousand years. Indigo naturalis is a combination of Chinese herbs known as Qing dai; it is traditionally used as an antipyretic, anti-inflammatory, antiviral and detoxifying drug [22]. Tryptanthrin (TRYP, Figure 1) or indolo [2,1-b] quinazoline-6,12-dione, is a substance found in Japanese indigo *Polygonum tinctorium* Aiton [23] and present in Indigo naturalis. It is reported that TRYP has anti-inflammatory, antimicrobial, antimalarial and anticancer effects, indicating its potential value as a medicine [24]. 

TRYP has been our focus for many years because of its promising effects. Recently, we reported the synthesis of TRYP by condensation of isatin and anthranilic acid using POCl_3_ as catalyst [25]. Single crystals of TRYP were obtained and its structure is shown in Figure 1. In our subsequent studies, TRYP was found to be effective in protecting mice against experimentally induced colitis by DSS. However, the concrete mechanism of TRYP exerting its protective role still needs further elucidation. The protein levels of two inflammatory mediators IL-6 and TNF-α were found to have changed in the group with TRYP treatment compared with the untreated colitis group. Actually, TNF-α and IL-6 are closely related to NF-κB and STAT3, respectively. After combining this evidence, we hypothesized that TRYP exerted its protecting effect against DSS induced colitis via regulating the TNF-α/NF-κB and IL-6/STAT3 signaling pathways. 

## 2. Results

### 2.1. TRYP Improves the Health Condition of Experimental Animals Suffered from DSS

**Figure 2:**
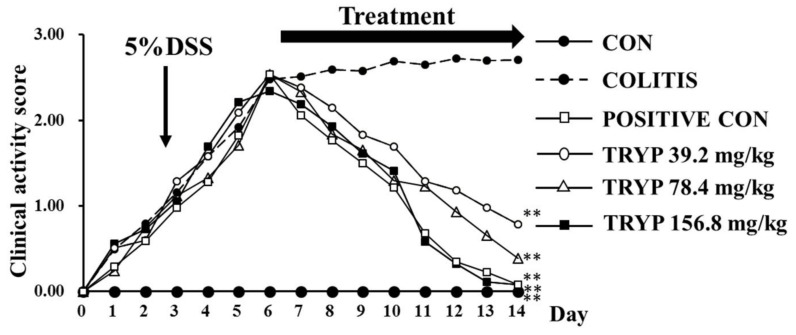
CAS of groups. After colitis induction, the animals were treated for 8 days with distilled water, DSS, sulfasalazine (“positive con”) and three concentrations of TRYP. Mice in colitis, positive control and TRYP groups were also given DSS during treatment. The untreated showed no significant improvement after 14 days while treatment with sulfasalazine and three concentrations of TRYP has significantly improved the CAS of colitis mice. *n* = 6, ** *p* < 0.01 compared with colitis.

Clinical activity score (CAS) was employed in our work to estimate the health status of the animals enrolled in our experiment. In addition, the CAS of each group could reflect the DSS mouse model and the therapeutic effect of positive drug and TRYP. The results of Figure 2 show the CAS of each group during 14 days. The line chart of the colitis group indicate that the colitis model was successful. After treatment with sulfasalazine and TRYP for 8 days, both the sulfasalazine (as “positive control”) group and the three TRYP groups had the CAS dramatically decreased, implying therapeutic effects of sulfasalazine and TRYP. The therapeutic effect of 156.8 mg/kg of TRYP was almost equally with the positive control (Figure 2). 

### 2.2. TRYP Decreases the Levels of IL-6 and TNF-α

**Figure 3:**
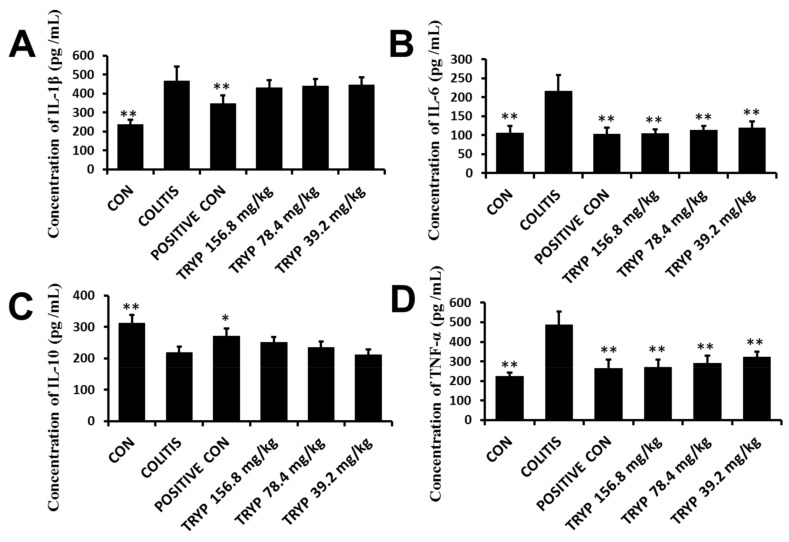
Levels of pro-inflammatory cytokines IL-1β, IL-6 and TNF-α and anti-inflammatory cytokines IL-10 in each group. (**A**) The concentration of IL-1β in each group; (**B**) The concentration of IL-6 in each group. After treatment with sulfasalazine and three concentration of TRYP, the levels of IL-6 decreased sharply; (**C**) The concentration of anti-inflammatory cytokines IL-10 in each group; (**D**) The concentration of TNF-α in each group. After treatment with sulfasalazine and three concentration of TRYP, the levels of TNF-α decreased sharply. Error bars represent means ± SEM of *n* = 6, * *p* < 0.05 and ** *p* < 0.01 compared with colitis.

Inflammatory cytokines play important roles in the occurrence and progression of colitis. The balance of pro-inflammatory cytokines and anti-inflammatory is essential for keeping a healthy condition in humans. In our experiment, four inflammatory cytokines were enrolled and as shown in Figure 3 the levels of pro-inflammatory cytokines IL-1β, IL-6 and TNF-α were all increased in colitis comparing with the negative control group. Contrarily, the level of anti-inflammatory cytokine IL-10 decreased (compared to the control group) in the colitis group. However, after treatment with sulfasalazine and TRYP, the levels of IL-6 and TNF-α decreased greatly comparing with the colitis group, while the levels of IL-1β and IL-10 changed insignificantly. Therefore, IL-6 and TNF-α were chosen to elucidate the molecular mechanism of the therapeutic role of TRYP.

### 2.3. TRYP Improves the Morphological Structure of Colitis

**Figure 4:**
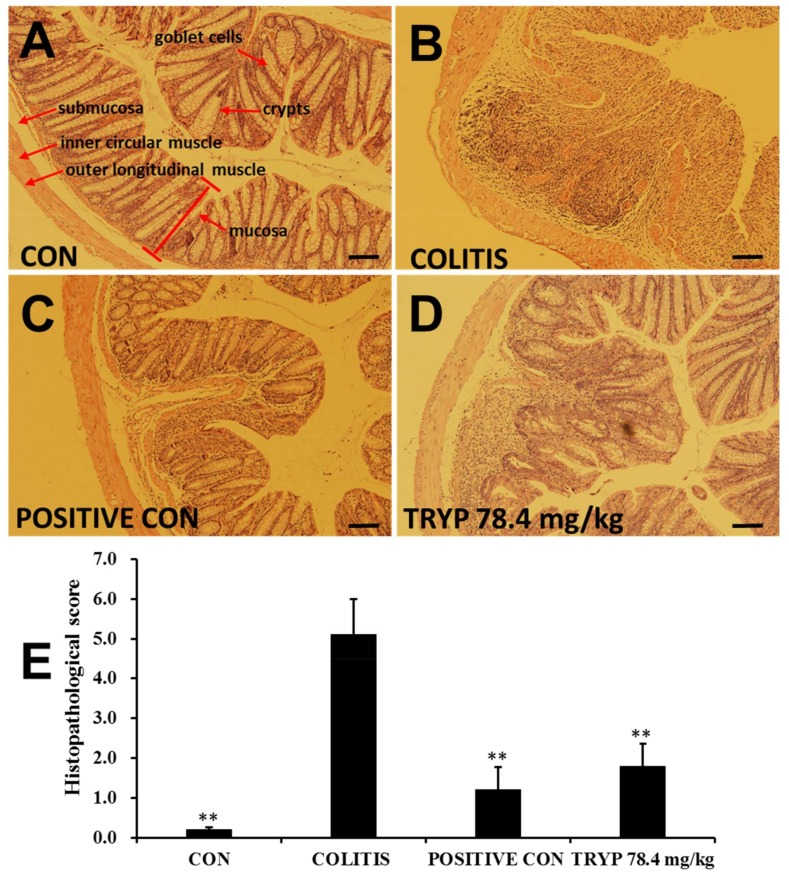
Histologic characteristics of each group, magnification ×100. (**A**) Normal colonic mucosa with regularly formed colonic folds covered by intact mucosa. (**B**) The histologic characteristics of colitis group. Inflammatory cell completely infiltrated to mucosa and submucosa. (**C**) The histologic characteristics of sulfasalazine control group. (**D**) The histologic characteristics of TRYP group with a concentration of 7.84 mg/kg. (**E**) The sum of histological score obtained from blinded histopathological analysis. Error bars represented means ± SEM of *n* = 3, ** *p* < 0.01 compared with colitis.

The results in Figure 4 exhibit the histologic characteristics of each group in our experiment. Blind histopathological analysis of the mid-colon was obtained after 14 days by H&E staining. The intact colon structure in control group (Figure 4A), including mucosa, submucosa, inner circular muscle and outer longitudinal muscle, was clearly visible, with the crypts and goblet cells regularly arranged. Figure 4B shows the histopathological structure of colitis induced by DSS. The crypts and goblet cells almost disappeared, revealing multiple erosive lesions and inflammatory cell infiltration composed mainly of macrophages with fewer lymphocytes. The inflammatory process was limited to the inner circular muscle. The results of Figure 4C display the effects of treatment by sulfasalazine. Though the structure of colon was intact, inflammatory cells were also observed. This result indicates that sulfasalazine with concentration of 125 mg/kg could ameliorate the colitis condition induced by DSS. However, the damage to colon tissues caused by inflammatory cell infiltration was not completely annihilated. The H&E staining results of Figure 4D illustrate the treatment effects of TRYP with concentration of 78.4 mg/kg. A lot of inflammatory cells were still observed in mucosa. And crypt branching, distortion and atrophy was also present. The location of some crypts were moved. This TRYP could improve colitis, but the effect was not sufficient comparing with sulfasalazine control. The columns in Figure 4E show the histopathological scores of each group. The column heights of sulfasalazine control and TRYP were considerably lower than that of colitis group, indicating the good therapeutic effect. 

### 2.4. Effects of TRYP on the Expression Level of NF-κBp65 and p-STAT3

**Figure 5:**
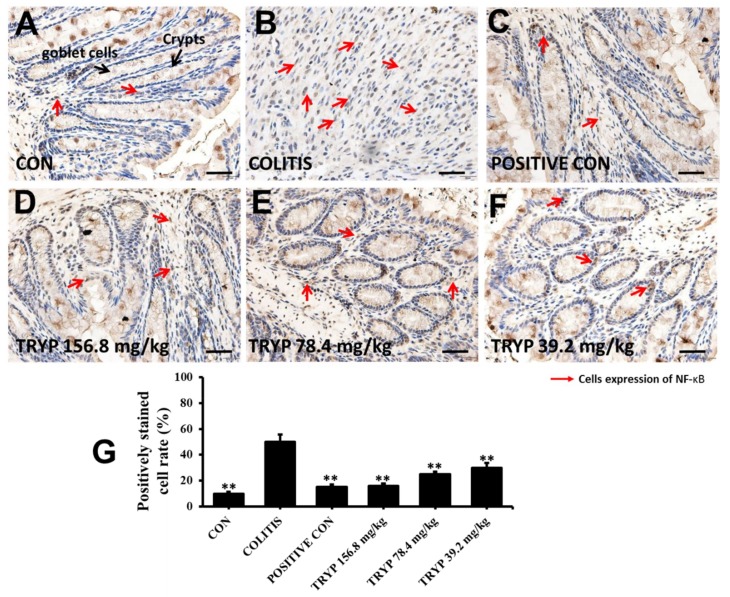
Administration with TRYP inhibited the expression of NF-κBp65, magnification ×400. (**A**) Immunohistochemistry result of control group with expressing NF-κBp65 barely. (**B**) The expression level of NF-κBp65 increased sharply with crypts and goblet cells disappeared. (**C**) Immunohistochemistry result of sulfasalazine control. (**D**–**F**) The expression level of NF-κBp65 with different concentrations of TRYP treatment. (**G**) The column heights represented the positively stained cell rates. Error bars represented means ± SEM of *n* = 3, ** *p* < 0.01 compared with colitis.

NF-κB and STAT3 are two key signaling proteins related to colitis. Therefore, the expression levels of NF-κBp65 and *p*-STAT3 were detected by immunohistochemistry. NF-κBp65 was located in cytoplasm normally at inactive conditions with IκBs bind. The results of Figure 5 show that the expression level of NF-κBp65 was increased considerably in the colitis group induced by DSS compared with control group, indicating the close relationship with colitis. The expression levels of NF-κBp65 were all decreased in sulfasalazine control and three concentrations of TRYP groups. Furthermore, the column height of TRYP with concentration of 156.8 mg/kg was almost equal to that of the sulfasalazine control.

**Figure 6:**
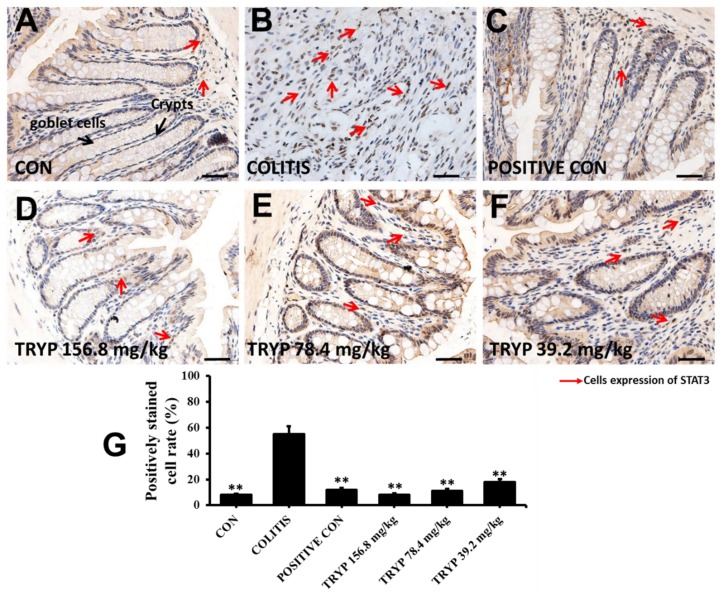
Treatment with TRYP suppressed the expression of *p*-STAT3, magnification ×400. (**A**) Immunohistochemistry result of control group with expressing *p*-STAT3 only. (**B**) The expression level of *p*-STAT3 increased sharply and both crypts and goblet cells disappeared. (**C**) Immunohistochemistry result of sulfasalazine control. (**D**–**F**) The expression level of *p*-STAT3 with different concentrations of TRYP treatment. (**G**) The column heights represented the positively stained cell rates. Error bars represented means ± SEM of *n* = 3, ** *p* < 0.01 compared with colitis.

STAT3 was another key signaling molecule that regulates cell circle, apoptosis and migration. Phosphorylated STAT3 (*p*-STAT3) can be transferred to the nucleus and exert there its regulatory functions. The expression levels of *p*-STAT3 in our experiment were changed similarly to that of NF-κBp65. The column height of TRYP at a concentration of 156.8 mg/kg was even lower than that of sulfasalazine control (Figure 6) 

### 2.5. TRYPIinhibits NF-κBp65 Transferring to Nucleus via Preventing Degradation of IκBα

**Figure 7:**
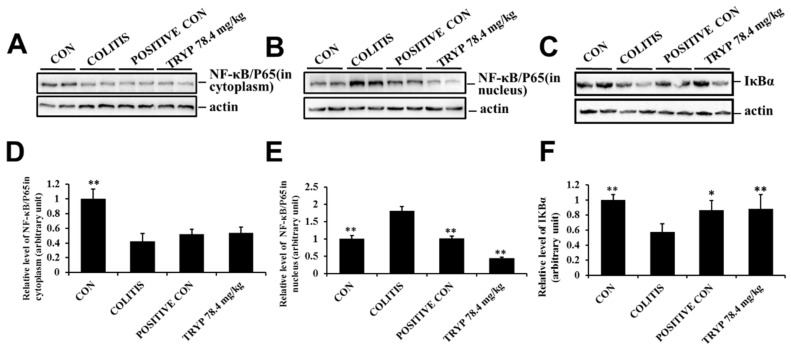
Effects of TRYP on the protein levels of NF-κBp65 and IκBα. (**A**) Results of TRYP with concentration of 78.4 mg/kg on the protein level of NF-κBp65 in cytoplasm; (**B**) The protein levels of NF-κBp65 in nucleus; (**C**) Effects of TRYP on the protein levels of IκBα; (**D**–**F**) The column charts represented the relative protein level of NF-κBp65 and IκBα. Error bars represented means ± SEM of *n* = 3, * *p* < 0.05 and ** *p* < 0.01 compared with colitis.

The expression level of NF-κBp65 was detected in our experiment to elucidate the role of NF-κBp65 in colitis. While NF-κBp65 was activated, it transferred to nucleus to exert its functions. Therefore, the protein levels of NF-κBp65 in cytoplasm and nucleus were all detected and the results showed that the protein level of NF-κBp65 in cytoplasm was decreased comparing with the control group. After administration of sulfasalazine and TRYP, the protein level was insignificantly increased. The variation curve of NF-κBp65 concentration in the nucleus was contrary to that in the cytoplasm. In addition, the protein level in TRYP groups was lower than that of the sulfasalazine control. The protein level of IκBα was also tested in our experiment and the results showed that the column height of colitis was lower than that of control, implying the activation of NF-κBp65. After treatment with sulfasalazine and TRYP, the protein levels of IκBα were much increased. Altogether, these results do indicate that NF-κBp65 is a key protein related to colitis and TRYP could inhibit its transfer to the nucleus via suppressing the degradation of IκBα (Figure 7).

### 2.6. TRYP Inhibits the Phosphorylation of STAT3

**Figure 8:**
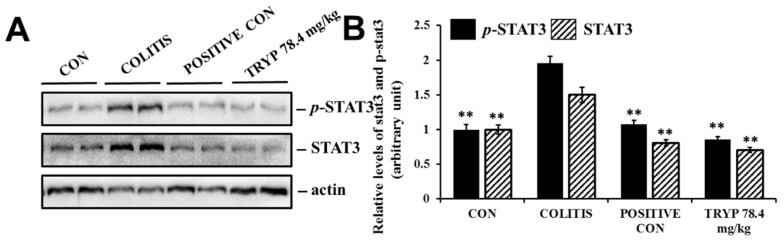
Effects of TRYP on the protein levels of STAT3 and *p*-STAT3. (**A**) Results of TRYP with concentration of 78.4 mg/kg on the protein level of stat and *p*-STAT3; (**B**) The column charts represented the relative protein level of stat and *p*-STAT3. Error bars represented means ± SEM of *n* = 3, ** *p* < 0.01 compared with colitis.

The expression level had been detected using immunohistochemistry. To elucidate the important roles in colitis, the protein level was also tested by western blot. The results showed that the protein levels of STAT3 and *p*-STAT3 were all enhanced in colitis comparing with the control group. After administration of sulfasalazine and TRYP, all protein levels decreased. These results indicate that STAT3 is a key protein in the progression of colitis, and TRYP may prevent the regulatory role of STAT3 in the nucleus by inhibiting its phosphorylation (Figure 8).

## 3. Discussion

IBD is a severe health problem that negatively affects the quality of human life. Although the roles of pathogenic factors of IBD are insufficiently understood, it is believed that inflammatory cells and cytokines play an important role in the occurrence and progression of IBD [26]. TRYP is an active constituent isolated from Japanese indigo (*Polygonum tinctorium* Aiton), what proved to have a protective effect on colitis experimentally induced by dextran sodium sulphate. Obviously, the concrete mechanisms need further investigation. 

The experimental results of H&E staining and CAS suggested that TRYP could improve the histopathological structure of colitis induced by DSS and the health conditions of the colitis mice. In our experiment, pro-inflammatory cytokines IL-6 and TNF-α were all significantly decreased after administration of TRYP. Therefore, NF-κB and STAT3, which were closely related with TNF-α and IL-6, were involved in our work to elucidate the concrete molecular mechanism of the protective effect of TRYP [27,28].

NF-κBp65 is normally located in cytoplasm, but once NF-κBp65 becomes activated by signaling transduction, it transfers to the nucleus to execute its functions. Therefore, the expression levels of NF-κBp65 in cytoplasm and nucleus always reflected the state of NF-κBp65. Our results showed that TRYP could regulated the protein level of NF-κBp65 to exert its therapeutic function. Furthermore, after administration of TRYP, the protein level of IκBα was increased, implying that TRYP could inhibit the degradation of IκBα to regulate the state of NF-κBp65. In conclusion, TRYP could regulated the NF-κBp65 levels by preventing the degradation of IκBα to exert its protective roles in mice with DSS induced colitis. Besides, the phosphorylation level of STAT3 was decreased after administration of TRYP, indicating the protective effect of TRYP on colitis was closely related with STAT3.

It was reported widely that NF-κB and STAT3 were two major transcriptional factors activated in colitis. The functionally interactions between them were also investigated at many different layers. It was reported that the members of NF-κB, such as RelA, could physiologically interact with STAT3 and their association could modify their transcriptional activity [29]. Besides, NF-κB and STAT3 could cooperatively bind at a subset of gene promoters to collaboratively induce their target genes expression [30]. Our results showed that the protective effect of TRYP on colitis was exerted through preventing NF-κB transfer to nucleus and the phosphorylation of STAT3. However, whether there were crosstalk between NF-κB and STAT3 and the association of NF-κB and STAT3 remains elusive. It was also reported that many cytokines like IL-6 induced by NF-κB or STAT3 can feedback to induceSTAT3 and NF-κB activation [31]. Although our work revealed that the expression levels of IL-6 and TNF-α and their corresponding signaling proteins NF-κB or STAT3 were changed greatly after treatment with TRYP. The feedback mechanisms remains need further investigation.

TRYP is a quinazoline alkaloid extracted from Indigo naturalis with a wide range of bioactivies, such as anti-inflammatory, antimicrobial, antimalarial and anticancer effects. In our work, the protective effect of TRYP on colitis has been confirmed. And the molecular mechanism was related with NF-κB and STAT3. However, the concrete molecular mechanisms remains to investigate. Besides, in the experiment to dissect mice to get colon, it was observed that lots of TRYP in colon. These results suggested that the intestinal absorption was not very good. Therefore, it is also deserved to investigate the active form of TRYP to exert its protective effect. 

In summary, colitis mice model were established to investigate the protective effect of TRYP on colitis. Our experiment results showed that TRYP could reduce the erosive lesions and inflammatory cell infiltration during colitis. The molecular mechanisms involved two key signaling molecules NF-κBp65 and STAT3. TRYP could affect the protein level of IκBα to regulate the expression of NF-κBp65 and the phosphorylation of STAT3. 

## 4. Materials and Methods 

### 4.1. DSS Mouse Models

Male SD mice weighting 20 ± 2 g were purchased from Laboratory Animal Center of Xi’an Jiao Tong University Health Science Center (Xi’an, China). Animals were maintained under standard laboratory conditions and were given autoclaved ultra-filtered water and animal feed ad libitum. Animal handling and scoring of colitis were performed in a consequently blinded experimental design. DSS (40,000 Da) was purchased from MP Biomedicals (Santa Ana, CA, USA) and dissolved in distilled water. Colitis was induced by providing drinking water containing 5% DSS for 6 days. Control mice received distilled water. Positive control mice were provided sulfasalazine with concentration of 125 mg/kg through intragastrical administration for another 8 days [32]. The other three groups were given TRYP twice a day with concentrations of 39.2 mg/kg, 78.4 mg/kg and 156.8 mg/kg for 8 days. All mice in colitis group, positive control and TRYP treatment group were given 5% DSS during in treatment [33].

### 4.2. CAS of Experiment Animals

A CAS involving animals’ body weight loss, stool consistency and rectal bleeding was recorded daily during the experiment as described previously [34]. The total clinical score was ranged from 0.0 (healthy) to 4.0 (maximal activity of colitis).

### 4.3. Cytokine Assays

The experimental animals were sacrificed after 14 days and eyeballs were excised to take blood. Subsequently, the blood was centrifuged to test the levels of IL-6, IL-10, IL-1β and TNF-α using ELISA kits according to the manufacturer’s instructions. ELISA kits of IL-6 and TNF-α were obtained from Neobioscience (Shenzhen, China; Batch Code, 201701). ELISA kits of IL-1β and IL-10 were purchased from Suzhou Calvin Biological Science and Technology Co. (Suzhou, China; Batch Code, 201611). 

### 4.4. Histopathological Evaluation

The extracted colon was spread onto a plastic sheet, fixed with 10% neutral-buffered formalin for 48 h, and embedded in paraffin block. Sections of paraffin-embedded tissues were subjected to hematoxylin & eosin (H&E) staining for measuring the severity of inflammation. Histologic scoring was performed by a pathologist. For infiltration of inflammatory cells, rare inflammatory cells in the lamina propria were counted as 0; increased numbers of inflammatory cells in the lamina propria as 1; confluence of inflammatory cells, extending into the submucosa as 2; and a score of 3 was given for transmural extension of the infiltrate. For tissue damage, no mucosal damage was counted as 0, discrete lymphoepithelial lesions were counted as 1, surface mucosal erosion was counted as 2, and a score of 3 was given for extensive mucosal damage and extension through deeper structures of the bowel wall. The combined histologic score ranged from 0 (no changes) to 6 (extensive cell infiltration and tissue damage).

### 4.5. Immunohistochemistry

Tissues sections for immunohistochemistry were prepared from formalin fixed, paraffin-embedded colon tissue. Stains against NF-κB p65 (CST, #8242S) and *p*-STAT3 (CST, #9145S) were performed according to the kit protocol (KGOS60, KeyGEN, Nanjing, China). The slides were briefly deparaffinized. Antigen unmasking was carried out by incubation in 100 °C water bath in 10 mM sodium citrate buffer with 0.1% Tween 20 for 20 min. Slides were incubated with primary antibodies. HRP Polymer was added and incubated at room temperature for 30 min. And the sections were stained with DAB substrate and counterstained with hematoxylin after 40 min. An isotype matched IgG served as the negative control for each immunostaining procedure. The density of NF-κBp65 and *p*-STAT3 were measured by using Image Pro Plus 6.0 software (Media Cybernetics, Silver Springs, MD, USA). The labeled inflammatory cytokines were automatic counting to quantify the experimental data.

### 4.6. Western Blot Analysis 

Tissues sections were grinded to homogenate in homogenizer and washed with ice-cold phosphate-buffered saline (PBS) and lysed using RIPA buffer supplemented with protease and phosphatase inhibitor mixtures (Heart Biological Technology Co., Xi’an, China) on ice. Lysates were separated by centrifugation at 4 °C and 14,000× *g* for 10 min. Protein concentration was determined by BCA assay (Thermo Fisher Scientific, Waltham, MA, USA). 50 mg total protein was subjected to sodium dodecyl sulfate-polyacrylamide gel electrophoresis (SDS-PAGE) and transferred to PVDF membranes (Millipore, Bedford, MA, USA). After blocking with 5% non-fat milk, the membranes were incubated with rabbit mAbs specific for p65-NF-κB (CST, #8242S), IκBα (CST, #4812S), STAT3 (CST, #12460S), *p*-STAT3 (CST, #9145S) overnight at 4 °C, next day the membranes were incubated with horseradish peroxidase conjugated secondary antibodies (1:3000) 37 °C for 1 h. Then immunoreactive proteins were visualized using ECL Western blotting detection reagent (Millipore, Billerica, MA, USA) and detected using Multi Image Light Cabinet Filter Positions (Alpha Innotech, San Leandro, CA, USA).

### 4.7. Statistical Analysis

Each experiment was repeated at least three times, and the data are presented as the mean ± SEM. Statistical differences between groups were analyzed by Student’s *t*-test or the Mann-Whitney U test as appropriate using a SPSS 13.0 program. * *p* < 0.05 was considered statistically significant.
