# Correction: Wang et al. Tryptanthrin Protects Mice against Dextran Sulfate Sodium-Induced Colitis through Inhibition of TNF-α/NF-κB and IL-6/STAT3 Pathways. *Molecules* 2018, *23*, 1062

**DOI:** 10.3390/molecules30234638

**Published:** 2025-12-03

**Authors:** Zheng Wang, Xue Wu, Cui-Ling Wang, Li Wang, Chen Sun, Dong-Bo Zhang, Jian-Li Liu, Yan-Ni Liang, Dong-Xin Tang, Zhi-Shu Tang

**Affiliations:** 1 Shaanxi Collaborative Innovation Center of Chinese Medicinal Resources Industrialization, Shaanxi University of Chinese Medicine, Xianyang 712083, China; 2 Shaanxi Province Key Laboratory of New Drugs and Chinese Medicine Foundation Research, Shaanxi University of Chinese Medicine, Xianyang 712083, China; 3 Shaanxi Rheumatism and Tumor Center of TCM Engineering Technology Research, Shaanxi University of Chinese Medicine, Xianyang 712083, China; 4 Key Laboratory of Resource Biology and Biotechnology in Western China, Ministry of Education, College of Life Science, Northwest University, Xi’an 710069, China; 5 Guizhou Province Hospital of Traditional Chinese Medicine, Guiyang University of Chinese Medicine, Guiyang 550002, China

## Error in Figure

In the original publication [1], there was a mistake in Figure 5 as published. Figure 5C,D showed high doses of the TYRP group. This was a mistake made during the process of organizing the results. The corrected Figure 5 appears below.

## Addition of Supplementary Materials

The original data of Figure 5G has been added under the Supplementary Materials based on the Academic Editor’s suggestions. This section is as follows:

**Supplementary Materials:** The following supporting information can be downloaded at https://www.mdpi.com/1420-3049/23/5/1062/s1.

The citation of it has been added in the caption of Figure 5G, as above.

The authors state that the scientific conclusions are unaffected. This correction was approved by the Academic Editor. The original publication has also been updated.

## Figures and Tables

**Figure 5:**
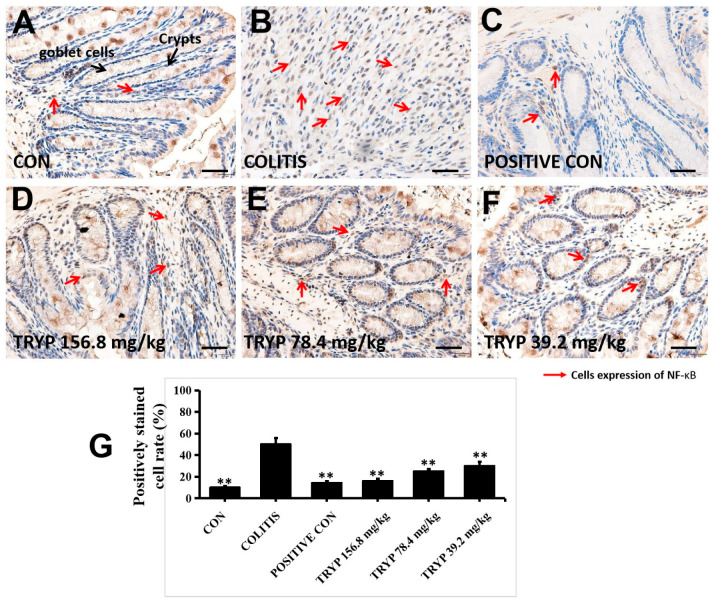
Administration with TRYP inhibited the expression of NF-κBp65, magnification ×400. (**A**) Immunohistochemistry result of control group with expressing NF-κBp65 barely. (**B**) The expression level of NF-κBp65 increased sharply with crypts and goblet cells disappeared. (**C**) Immunohistochemistry result of sulfasalazine control. (**D**–**F**) The expression level of NF-κBp65 with different concentrations of TRYP treatment. (**G**) The column heights represented the positively stained cell rates. Error bars represented means ± SEM of *n* = 3, ** *p* < 0.01 compared with colitis (see Supplementary Materials).
